# Controlled Growth of Oligophenylene‐Structures on Graphene for Facile Secondary Functionalization

**DOI:** 10.1002/anie.202504482

**Published:** 2025-05-10

**Authors:** Christian E. Halbig, Felix Fels, Shenquan Wei, Robert Schusterbauer, Ievgen Donskyi, Markus R. Heinrich, Siegfried Eigler

**Affiliations:** ^1^ Freie Universität Berlin Altensteinstraße 23a 14105 Berlin Germany; ^2^ Department Chemie und Pharmazie Pharmazeutische Chemie Friedrich‐Alexander‐Universität Erlangen‐Nürnberg Nikolaus‐Fiebiger‐Str. 10 91058 Erlangen Germany

**Keywords:** Functionalization, Graphene, Oligophenylene, Polyphenylene, Surface chemistry

## Abstract

Functionalization of graphene derivatives is a common approach to tune material properties for use in various applications. Because of the low reactivity of the unsaturated carbon lattice of graphene, not only are few chemical approaches suitable for successful functionalization, such as those involving highly reactive in situ formed radical species or nitrene and carbene compounds, but also the degree of functionalization is usually limited, modifying only a few percent of the carbon atoms. Typically, uncontrolled side reactions such as homocoupling and oligomerization of newly introduced functional groups can occur instead of direct coupling to the carbon lattice. We want to turn this unwanted side reaction into an advantage and use intentionally formed covalent dendrimeric oligophenylene structures for secondary functionalization. We show that these oligomeric structures can be grown to specific thicknesses and used for further functionalization with bromomethyl groups at high density on the surface. This functionalization opens further avenues for subsequent nucleophilic substitution, as exemplified by the introduction of versatile azide, nitrile, and phosphonate groups. The results presented here are not only applicable to large oligophenylene structures, but also demonstrate that, in principle, single aryl moieties on graphene of any size and density can be successfully functionalized.

## Graphical abstract

By thermal decomposition of different BPO‐based peroxides with different substituents, dendrimeric oligophenylene structures with thicknesses up to 10 nm have been grown on graphene in a controlled manner. These newly introduced structures provide active sites for further functionalization as they have a higher reactivity than the graphene lattice, as shown by their subsequent functionalization with bromomethyl groups. These functional oligophenylene structures can serve as a versatile platform for secondary functionalization, as shown by the introduction of azide, nitrile, and phosphonate groups. The approach presented here opens up a novel route to generate nanometer‐sized graphene‐based materials with tailored properties for subsequent applications.

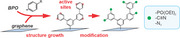

## Introduction

Covalent functionalization of graphene‐based materials is a commonly used method to tailor the properties for the desired application, which can range from use in (nano)filtration in membranes,^[^ ^1^, ^2^ ^]^ for drug delivery for biomedical treatment,^[^ ^3^, ^4^ ^]^ as additives in materials to increase thermal and mechanical strength,^[^ ^5^ ^]^ or in coatings, sensors, electronics, and more. The main limiting factor is the extraordinary chemical stability of the unsaturated sp^2^ carbon backbone and thus, most functionalization techniques suffer from restrictions, as low degrees of functionalization.^[^ ^6^, ^7^ ^]^ For the formation of C─C, C─N, or C─O bonds on the carbon lattice of graphene, highly reactive reactants such as radicals,^[^ ^8^, ^9^, ^10^ ^]^ electron‐deficient carbene and nitrene species,^[^ ^11^, ^12^ ^]^ or harshest oxidative reaction conditions are required.^[^ ^13^, ^14^, ^15^, ^16^ ^]^ In particular, C─C bond formation to graphene is suitable for grafting chemically stable out‐of‐plane functional motifs onto graphene using aryl radicals formed by decomposition of diazonium compounds,^[^ ^17^, ^18^, ^19^ ^]^ or with specific alkyl and aryl halides via reductive carbon–halogen bond cleavage.^[^ ^8^, ^20^ ^]^ Nevertheless, the degree of functionalization with respect to carbon atoms is usually limited to few percent.^[^ ^8^, ^21^, ^22^ ^]^ Furthermore, the radical intermediates are prone to side reactions such as H‐abstraction, homocoupling, and oligomerization, especially when the substituents are bulky or contain heteroatoms, or reactive sites are not protected by steric hinderance.^[^ ^19^, ^23^ ^]^ Thus, it is challenging to introduce complex functional motifs such as pharmaceutical relevant drugs or receptors at higher density on the surface of graphene by radical reactions.

**Scheme 1:**
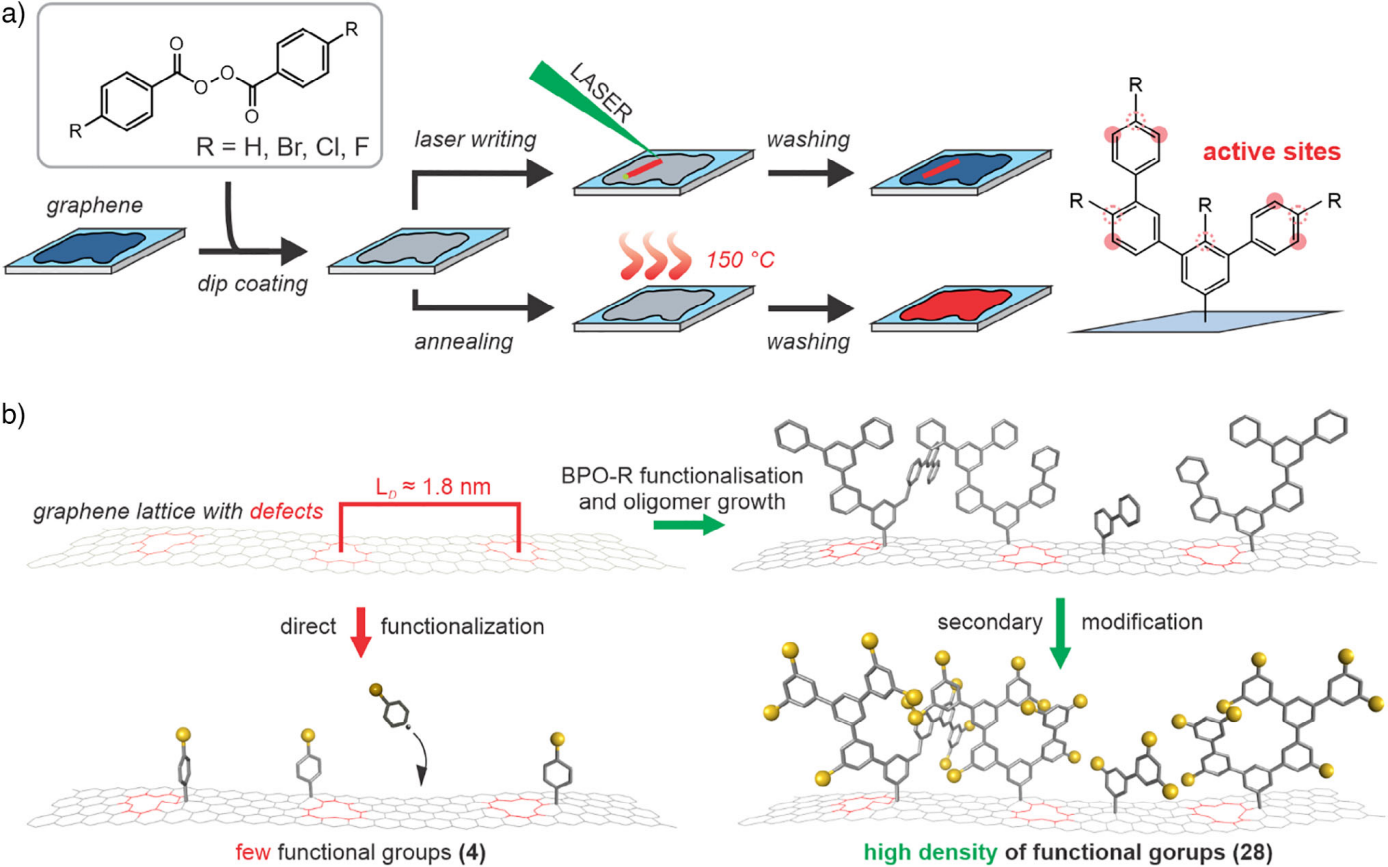
a) Process of functionalization of graphene on a 300 nm SiO_2_/Si wafer with dendrimeric oligophenylene chains by thermal decomposition (hotplate or laser) of benzoyl peroxide derivatives. The structure on the right side illustrates exemplary one oligophenylene branch on the graphene lattice. Reactive sites in *meta*‐position of the oligophenylene chain are marked with red dots. b) Advantage of the two‐step functionalization of graphene with dendrimeric oligophenylene structures in comparison to conventional direct functionalization. Yellow spheres indicate arbitrary functional groups.

Herein, we present a novel approach by exploiting controlled growth of dendrimeric oligophenylene branches on graphene and their subsequent chemical modification that allows postfunctionalization of graphene with a high density of functional groups (Scheme 1). These structures are formed by either laser or heat‐induced decomposition of adsorbed dibenzoyl peroxide derivatives on the surface of graphene. A part of the formed radicals binds to the graphene lattice and provides two new active sites in preferably *meta‐*position for chain growth and branching, or later secondary functionalization. In this study, based on atomic force microscopy images (AFM) and statistical analysis of Raman spectra, we show that tree‐like structures with phenyl groups are formed on the surface of graphene. Thereby, phenyl groups are more reactive than the underlying carbon lattice and thus, a wide range of novel structural motifs can be easily attached to the nanomaterial via chemical reactions targeting the phenyl moieties. We demonstrate the versatility of this approach by introducing bromomethyl groups, which were subsequently modified on the surface by sodium azide, potassium cyanide, or triethylphosphite. Although the newly introduced nitrile and azide groups could be converted to carbonyl, carboxyl, or amine and amide groups^[^ ^24^ ^]^ or used later for click chemistry.^[^ ^25^, ^26^ ^]^ Phosphonates are highly versatile and could also act as a binding site for a wide range of metal ions and thus regulate bone function in pharmaceutical relevant drugs.^[^ ^27^, ^28^ ^]^

## Results and Discussion

### Formation of Dendrimeric Structures on Graphene

**Figure 1:**
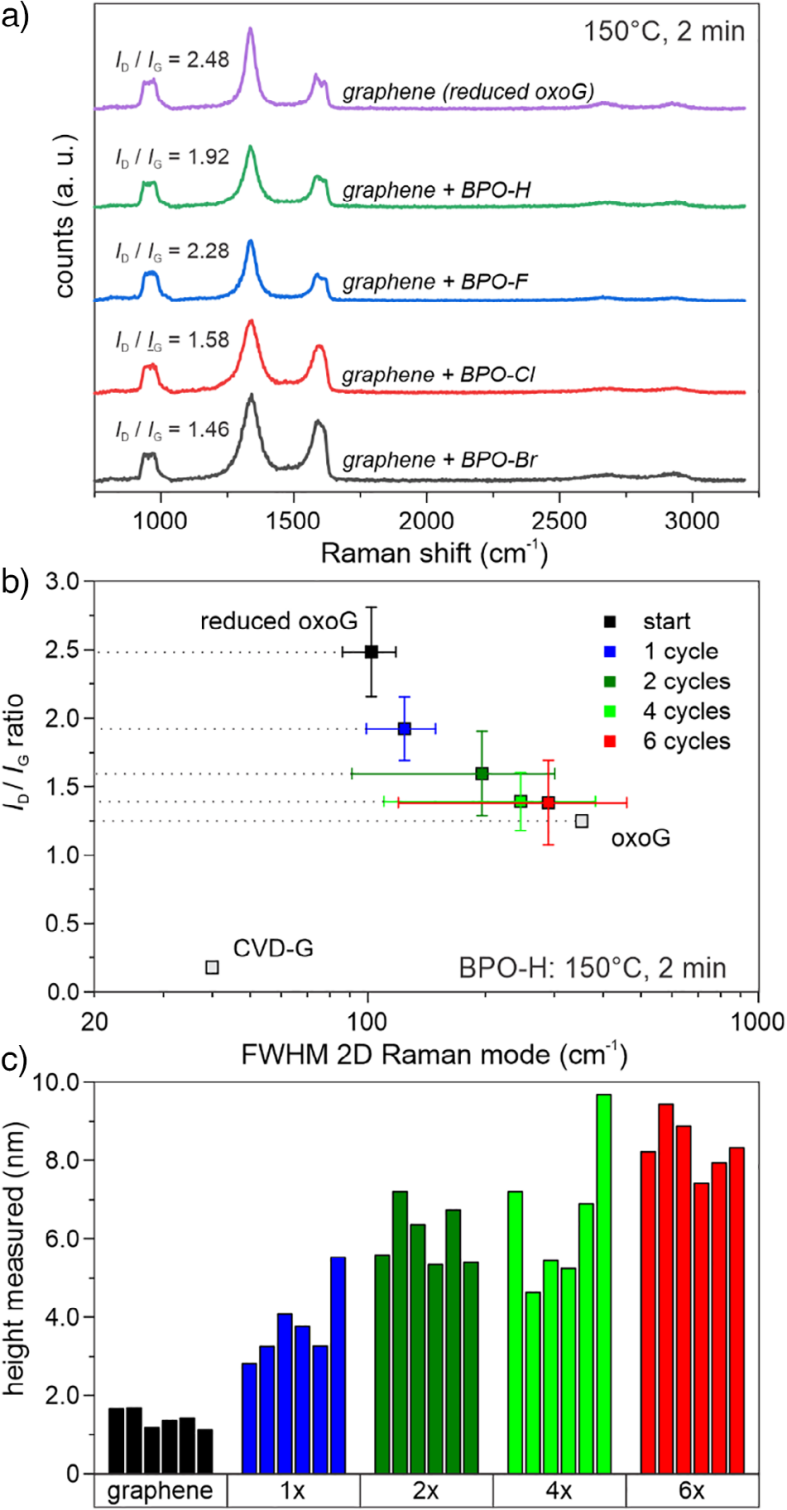
a) Representative Raman spectra of graphene samples before and after functionalization for one time with BPO‐R. The spectra were extracted from recorded Raman maps. b) Statistical Raman analysis of graphene films after up to six cycles of functionalization with BPO‐H under similar conditions. c) Measured heights from atomic force microscope images of functionalized graphene flakes after up to six cycles of functionalization with BPO‐H.

For most of our experiments, we used wet‐chemically prepared graphene with a low density of lattice defects. It was prepared by oxidation of graphite under controlled conditions at low temperature (<10 °C) with potassium permanganate in sulfuric acid to avoid excessive lattice defect formation by overoxidation of the carbon framework.^[^ ^29^ ^]^ Intermediary formed oxo‐functionalized graphene (oxoG, Figure S1) was deposited on 300 nm SiO_2_/Si substrates by Langmuir–Blodget technique, and subsequently reduced by hot vapor of hydrogen iodide and trifluoro acetic acid.^[^ ^30^ ^]^ Recorded Raman spectra revealed that the prepared graphene had a very low density of permanent lattice defects (*I*_D_/*I*_G_ ratio = 2.48 ± 0.33, Figure 1a). For other experiments, where an even lower density of defects is beneficial, we used almost intact graphene prepared from chemical vapor deposition on copper foil (CVD, *I*_D_/*I*_G_ ratio <0.2).

For the generation of oligophenylene structures on graphene, different dibenzoyl peroxide derivatives were applied on the surface of the two graphene samples by dipping of the SiO_2_/Si substrates into strongly diluted solutions of the corresponding compounds in acetone (∼5 mM). The remaining thin solvent film evaporated under ambient conditions within few seconds. We tested several peroxides, comprising regular dibenzoyl peroxide (BPO‐H) and its halogenated derivatives bearing Br, Cl, or F atoms in *para*‐position on each benzene ring (BPO‐Br, BPO‐Cl, and BPO‐F). Functionalization was achieved by placing the coated substrates on a hotplate at 150 °C for 2 min to induce aryl radical formation by thermal decomposition of the peroxides, or controlled irradiation of the sample surface with a 532 nm laser (Scheme 1). The thermal process was repeated up to six times to obtain reliable statistical Raman data and determine efficiency of the reactants.^[^ ^31^ ^]^ The annealing process of graphene coated surfaces is suitable to functionalize large areas within short time, but laser‐induced functionalization can be used to write specific patterns at specific irradiation energies on surfaces giving control on the structure. We applied this concept on low‐defective CVD‐graphene (Figure S2).

We clearly observed that the graphene lattice becomes successively covalently functionalized with each repetition of coating and upheating, as indicated by a drop of the *I*_D_/*I*_G_‐ratio of the graphene in the Raman spectra. Accompanied by this, the full‐width‐at‐half‐maximum (FWHM) of the main Raman modes (D, G, and 2D) and absolute measured intensity of the G mode for a single graphene layer increased with respect to the multiphonon scattering band from the silicon substrate between ∼930 and1030 cm^−1^ (Figure 1a). This phenomenon goes hand in hand with various reports from literature, and doesn't occur in the absence of BPO derivatives (Figure S1c,d).^[^ ^8^, ^32^, ^33^ ^]^ Interestingly, the different peroxides have markedly different reactivity. Already after the first coating and heating cycle with BPO‐Br, the Raman spectrum of the functionalized graphene had already a line shape similar to that of highly functionalized oxoG, while at least six cycles were necessary for BPO‐H and BPO‐F to achieve a comparable degree of functionalization (Figure S3). The trend of reactivity in declining order clearly follows the trend:

Br>CL>H>F



As seen for CVD‐Graphene, the alteration of the *I*_D_/*I*_G_ ratio originates from functionalization of the carbon lattice with phenyl moieties (sp^3^ defect formation) as the process is reversible under elevated temperature due to high energy irradiation (Figure S2).^[^ ^8^ ^]^ Concerning the different reactivities, we assume that the observed trend in reactivity of the peroxides could originate from the different volatility of the formed radical species. The boiling points of benzene and fluorobenzene are 80 and 85 °C, while the heavier chlorobenzene and bromobenzene boil at much higher temperatures of 135 and 156 °C, respectively. According to this assumption, one might expect that the degree of functionalization reached by BPO‐F is higher than that of BPO‐H. However, the small disadvantage of BPO‐H arising from the lower boiling point of benzene is likely to be counterbalanced by additional free *para*‐position on the aromatic core allowing more effective oligomerization.

After each functionalization cycle of graphene, the corresponding heights of several individual flakes were extracted from the corresponding recorded AFM images (Figure 1c, S4–S6). For the BPO‐H functionalization series, a single layer of the initial graphene flakes after reduction of oxoG had an initial particle thickness of approximately 1.7 nm. After the first cycle, the thickness of the particles slightly increased to values between 2.8 and 5.5 nm, until a height of 7.4–9.4 nm is reached after the sixth cycle. We have calculated that for an ideally arranged, vertical network of benzene rings in *para*‐position, 8 benzene units would add about 2.3 nm to the flake thickness. Accordingly, the 5.7–7.7 nm increase in thickness from six cycles of BPO‐H functionalization would result in oligophenylene chains with 20 benzene rings, but it is highly likely that this number is actually exceeded due to imperfect and inhomogeneous growth in random direction. The measured thickness of individual flakes had a certain variation, most likely due to the manual dip coating and subsequent uncontrolled evaporation of remaining acetone. This may lead to a coating of inhomogeneous thickness on different spots on the substrate, analogously to the so called “coffee‐ring effect”.^[^ ^34^ ^]^ Nevertheless, a more controlled growth of the oligophenylene chains seems feasible under further optimized conditions. When comparing the formation of the oligophenylene structures on graphene with the other peroxides the trend differs from the reactivities for the functionalization of the graphene lattice (Figure S2)

H≈Br>CL>F



**Figure 2:**
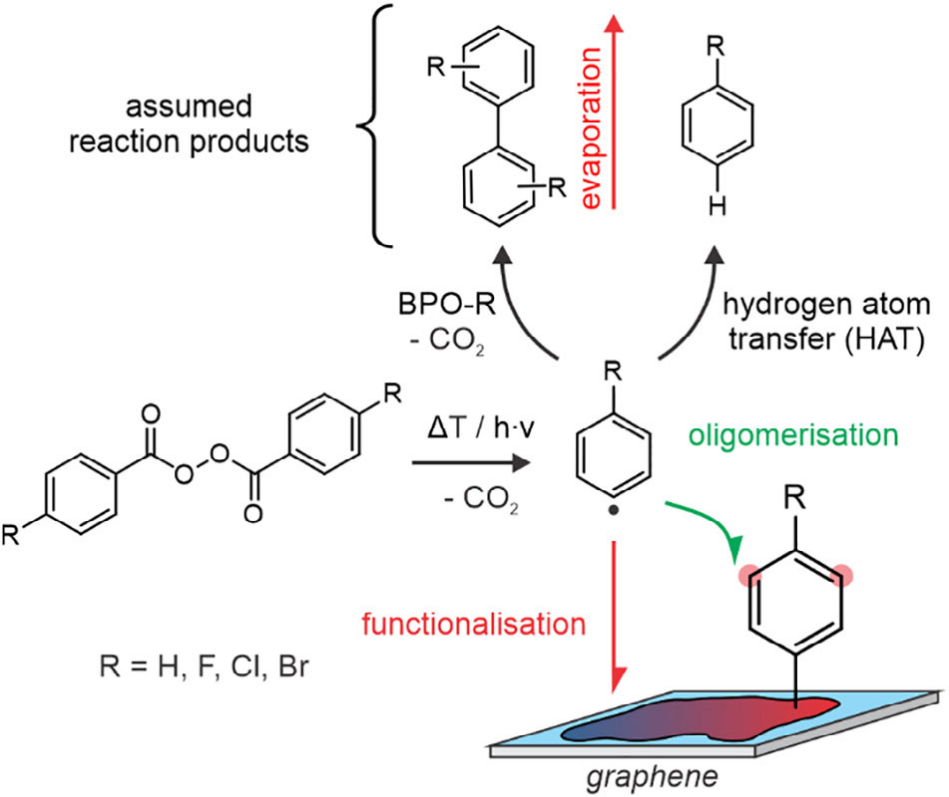
Sketch illustrating all possible events after aryl radicals are formed in proximity of a graphene flake at elevated temperatures.^[^ ^38^, ^39^ ^]^

It is not surprising that direct graphene surface functionalization and oligomerization occur in parallel with different reaction rates (Figure 1b,c) and that already present oligophenylene does not completely passivate the remaining unfunctionalized graphene surface toward a further aryl radical attack. Although BPO‐H has a quite poor reactivity toward the graphene lattice, it is able to efficiently generate dendrimeric structures on the surface similar to BPO‐Br. We used the empirical formula of Lucchese and Cançado et al. to estimate the degree of functionalization basing on the average distance between two defects L_D_.^[^ ^35^, ^36^ ^]^ Based on the *I*_D_/*I*_G_‐ratio of the recorded Raman spectra, the average distance between defects (*L*_D_) in the initial graphene was ∼1.9 nm,^[^ ^35^, ^36^ ^]^ and decreased with each cycle of functionalization (Figures 1b, S3; 1st cycle: *L*_D_ = 1.64 nm / *I*_D_/*I*_G_‐ = 1.91; 2nd cycle: *L*_D_ = 1.53 nm / *I*_D_/*I*_G_‐ = 1.60). As clustering of multiple functional groups typically occurs around small‐sized activated domains – for instance around point defects, vacancies, or other functional groups,^[^ ^9^, ^32^ ^]^ the used empirical model cannot provide exact information about the absolute density of introduced functional groups, however, reported values for materials with similar Raman spectra are between 1% and 2% with respect to carbon atoms.^[^ ^8^, ^37^ ^]^ Nevertheless, the calculated *L*_D_ values indicate that there are sufficient unfunctionalized sp^2^ carbon atoms left between the defective domains and theoretically accessibly yet larger oligophenylene structures are favorably formed. As the used cantilever has a tip‐size of around 10 nm, exceeding the *L*_D_ of the initial graphene already by the factor of 5, these unfunctionalized domains cannot be resolved, but we strongly assume that the formed structures may reflect more likely a forest‐like structure with large oligophenylene‐trees than a perfect homogeneous coating. This would be advantageous because it would increase the surface area of the rigid oligophenylene structures with their limited flexibility and solvent interaction. Thus, more phenyl rings can be addressed by subsequent secondary functionalization. Allover, all four competing processes occur during the functionalization of graphene with BPO‐H, namely functionalization with measurable defect formation, as well as chain‐growth, homocoupling, and evaporation–all without defect formation (Figure 2).

### Functionalization of the Oligophenylene‐Structures

**Figure 3:**
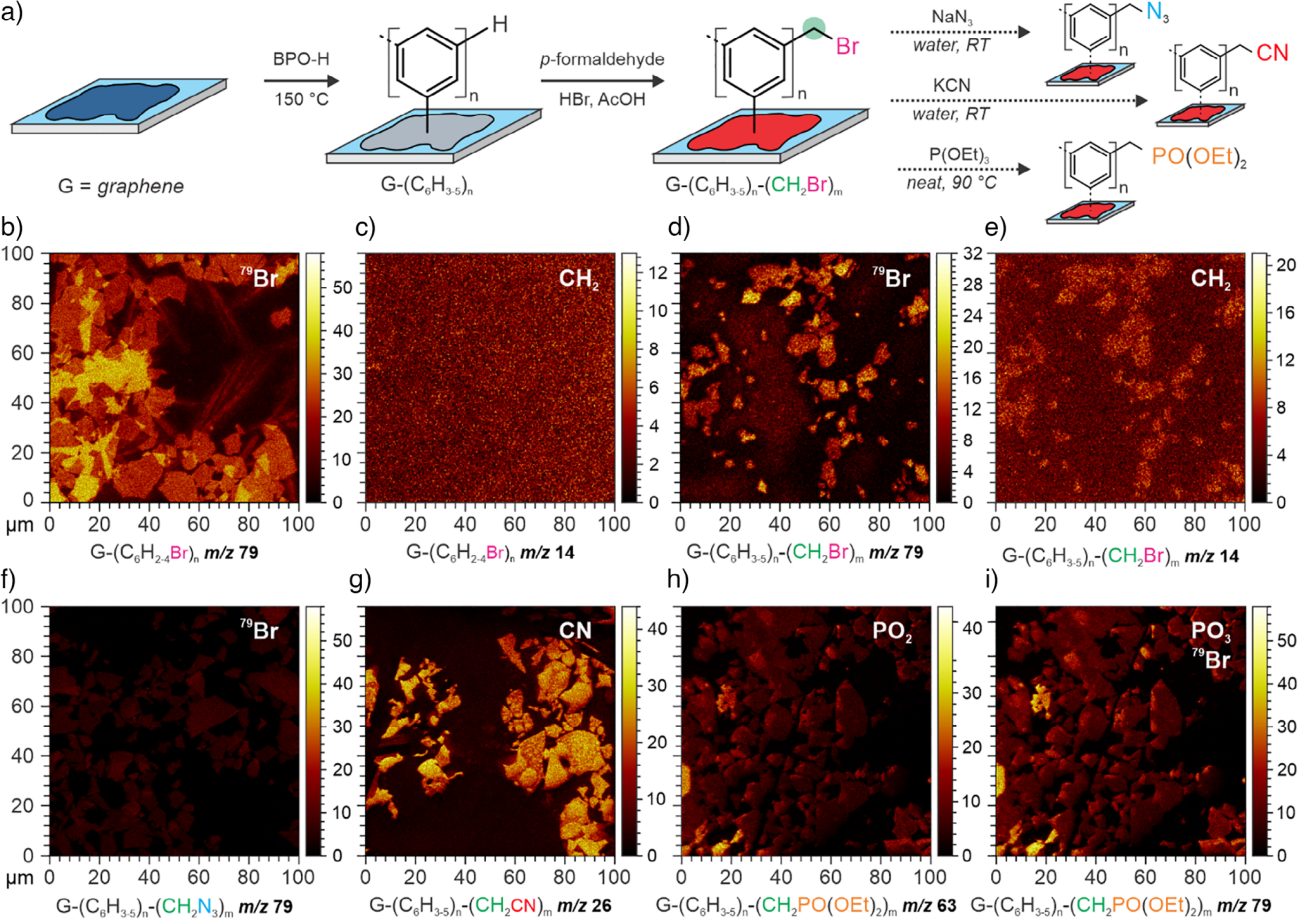
a) Reaction scheme to functionalize graphene with polyphenylene groups (G‐(C_6_H_3‐5_)_n_) and subsequent secondary functionalization with bromomethyl groups (G‐(C_6_H_3‐5_)_n_‐(CH_2_Br)_m_), followed by a Michaelis–Arbuzow reaction to introduce phosphonates. For the sake of clarity, only one representative phenyl group is shown and the dashed line represents the continuing chain. b)–i) ToF‐SIMS images of graphene with dendrimeric oligophenylene structures and subsequent secondary functionalization. The corresponding fragments used for imaging are shown below the images. b), c) Images of a graphene film functionalized solely with BPO‐Br (reference sample) and d), e) graphene after functionalization with BPO‐H and subsequent bromomethylation. f) Bromomethylated samples after further modification with aqueous sodium azide, g) aqueous potassium cyanide and h), i) triethylphosphite.

We have demonstrated that thermal generation of phenyl radicals (Figure 1) leads to a controlled formation of dendrimeric oligophenylene structures of graphene. In the following experiments, we used this structural covalent motif on graphene for further functionalization by introducing bromomethyl groups on the individual benzene rings by dipping the substrates with BPO‐H functionalized graphene particles (G‐(C_6_H_3‐6_)_n_) into a reaction mixture containing hydrogen bromide in glacial acetic acid and *para*‐formaldehyde, as previously reported for common aromatic compounds.^[^ ^40^ ^]^ In a second subsequent reaction step, azide, nitrile and phosphonate groups were introduced by nucleophilic substitution of the benzylic bromine atom (Figure 3a).

The success of the functionalization of the oligophenylene side chains was verified by time‐of‐flight secondary ion mass spectrometry (ToF‐SIMS) and X‐ray photoelectron spectroscopy (XPS). After bromomethylation of G‐(C_6_H_3‐5_)_n_, distinct new mass fragments of bromine with *m/z* 79 and 81 arise in the in the mass spectrum of thus obtained G‐(C_6_H_3‐5_)_n‐_(CH_2_Br)_m_ (Figure 3d) similar to BPO‐Br functionalized graphene G‐(C_6_H_2‐4_Br)_n_ (Figure 3b), however, the signals are significantly weaker. In contrast to G‐(C_6_H_2‐4_Br)_n_, an additional signal for methylene groups with *m/z* 14 is present in the ToF‐SIMS images of G‐(C_6_H_3‐5_)_n‐_(CH_2_Br)_m_ (Figure 3c,e). We can also find clear signals from emitted photoelectrons of Br in the XPS survey spectra at around 71 eV (Br 3d), 183 eV (Br 3p^3/2^), and 189 eV (Br 3p^1/2^). The low signal intensity shows that only the surface of the oligophenylene structures is functionalized, which is not surprising, as polyphenylenes are believed to be insoluble in any solvent, and thus, a reaction partner cannot easily enter the space between the individual branches.^[^ ^41^ ^]^ For approximately 10 nm thick oligophenylene structures on graphene, we determined from the XPS survey spectra an approximate ratio of one bromine atom on 30 to 50 carbon atoms, whereas all the bromine can only be present on the surface of the oligophenylene structures. Considering that 95% of the information from the recorded photoelectrons originate from surface near nuclei within the first 10 nm of the probed material,^[^ ^42^ ^]^ it seems plausible that the density of bromomethyl groups on the surface is approximately one order of magnitude higher, as unfunctionalized oligophenylene structures were also recorded.

**Figure 4:**
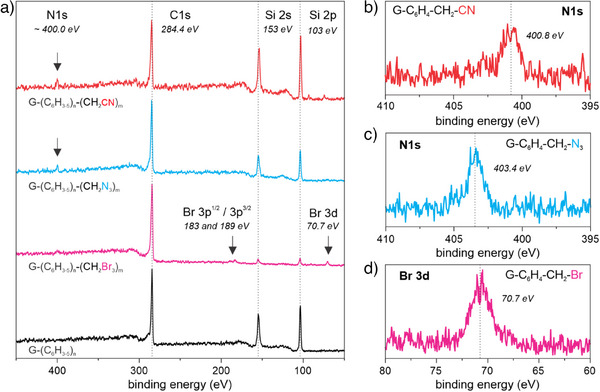
a) XPS survey spectra of graphene flakes on 300 nm SiO_2_/Si wafer, functionalized with BPO‐H (G‐(C_6_H_3‐5_)_n_, black) and after bromomethylation (G‐(C_6_H_3‐5_)_n_‐(CH_2_Br)_m_, purple) and subsequent nucleophilic substitution of the latter mentioned functional group with sodium azide (G‐(C_6_H_3‐5_)_n_‐(CH_2_N_3_)_m_, blue) and potassium cyanide (G‐(C_6_H_3‐5_)_n_‐(CH_2_CN)_m_, red). b)–d) High resolution spectra of the elements of interest in the newly introduced functional group on the oligophenylene structure with respect to the corresponding spectra survey spectra on the left.

After films of G‐(C_6_H_3‐5_)_n‐_(CH_2_Br)_m_ were immersed in aqueous solutions of sodium azide and potassium cyanide, the aforementioned bromine signals vanish while new signals at 400.8 and 403.4 eV arise for nitrile and azide groups, respectively (Figure 4).^[^ ^43^ ^]^ We would have expected a double signal for the azide group, however, azides are known to rapidly degrade under X‐ray irradiation.^[^ ^44^ ^]^ As a reference, we prepared azide functionalized oxoG by mixing the aqueous dispersion of the nanomaterial with sodium azide and subsequent freeze drying.^[^ ^45^ ^]^ In the corresponding XPS spectra of this material we can find the typical double signal for azide moieties at 400.0 and 403.4 eV (Figure S7). It seems that the different binding situation of the azide moiety –on a tertiary carbon atom on the graphene surface or in benzylic position on the oligomeric structure–is responsible for different stabilities of this moiety. Undoubtedly, this point needs more clarification in future work.

A successful substitution of the benzylbromides with triethylphosphite could not be directly verified by XPS as we didn't observe signals for phosphorous in the survey spectra of corresponding samples at around 134 eV (P 2p^3/2^) or 190 eV (P 2 s), however, the former signal for bromine is not detectable anymore in the recorded spectra. Nevertheless, clear signals for phosphorous containing fragments with *m/z* 63 and 79 for PO_2_ and Br/PO_3_ arise in the ToF‐SIMS spectra and images, respectively. With a quantitative look at the ToF‐SIMS spectra we can see that initial bromine signals *m/z* 79 and 81 have a very high intensity. After substitution with either azide, cyanide or triethylphoshite, the signals mostly vanish but are still visible.

Altogether we observed that all three substitution reactions are possible on surface bound oligophenylene functionalized graphene carrying bromomethyl groups. In the future, we plan to transfer this model reactions on bulk‐ graphene in dispersion to obtain large amounts of functionalized materials for later application in diverse fields.

## Conclusion

It has been demonstrated that covalently bonded oligophenylene‐based dendrimeric structures can be grown on graphene in a controlled manner using different dibenzoyl peroxide derivatives. Regular dibenzoyl peroxide and its halogenated derivatives showed different reactivity toward the graphene lattice and its dendrmeric structures, as evidenced by statistical Raman spectroscopy. Finally, the newly formed dendrimers can be easily functionalized with bromomethyl groups and further modified by common nucleophiles as demonstrated for azide, cyanide, and triethylphosphite. The introduction of bromomethyl groups opens up subsequent reactions, which have been verified by powerful surface analytical techniques, namely ToF‐SIMS and XPS. Our data indicate that, in principle, a wide range of phenyl moieties bound to the graphene lattice can be successfully functionalized, even at low concentrations below the detection limit of common surface and material analytical techniques. This route of modification of graphene‐based materials may serve in the future as a versatile platform for tuning the properties of graphene for any other subsequent application.

## Supporting Information

The authors have cited additional references within the Supporting Information.^[^ ^46^, ^47^, ^48^ ^]^

## Conflict of Interests

The authors declare no conflict of interest.

## Supporting information



Supporting Information

## Data Availability

The data that support the findings of this study are available in the Supporting Information of this article.
