# Supplementary material for: Controlled Growth of Oligophenylene‐Structures on Graphene for Facile Secondary Functionalization
Source: Angew Chem Int Ed Engl. 2025 May 10;64(25):e202504482. doi: 10.1002/anie.202504482 (PMC12171345; doi:10.1002/anie.202504482)
Supplement: Supplementary file 1 — Supporting Information [file ANIE-64-e202504482-s001.docx]

**Controlled Growth of Oligophenylene-Structures on Graphene for Facile Secondary functionalization.**

Christian E. Halbig^[a]‡^*, Felix Fels^[b]‡^, Shenquan Wei^[a]^, Robert Schusterbauer^[a]^, Ievgen Donskyi^[a]^, Markus R. Heinrich^[b]^* and Siegfried Eigler^[a]^

[a] Dr. C. E. Halbig, S. Wei, R. Schusterbauer, Dr. I. Donskyi, Dr. S. Eigler
SupraFAB,
Freie Universität Berlin
Altensteinstraße 23a, 14105 Berlin, Germany.
E-mail: christian.halbig@fu-berlin.de

[b] F. Fels, M. R. Heinrich
Department Chemie und Pharmazie, Pharmazeutische Chemie
Friedrich-Alexander-Universität Erlangen-Nürnberg
Nikolaus-Fiebiger-Str. 10, 91058 Erlangen, Germany
E-mail: markus.heinrich@fau.de

**Supporting Information**


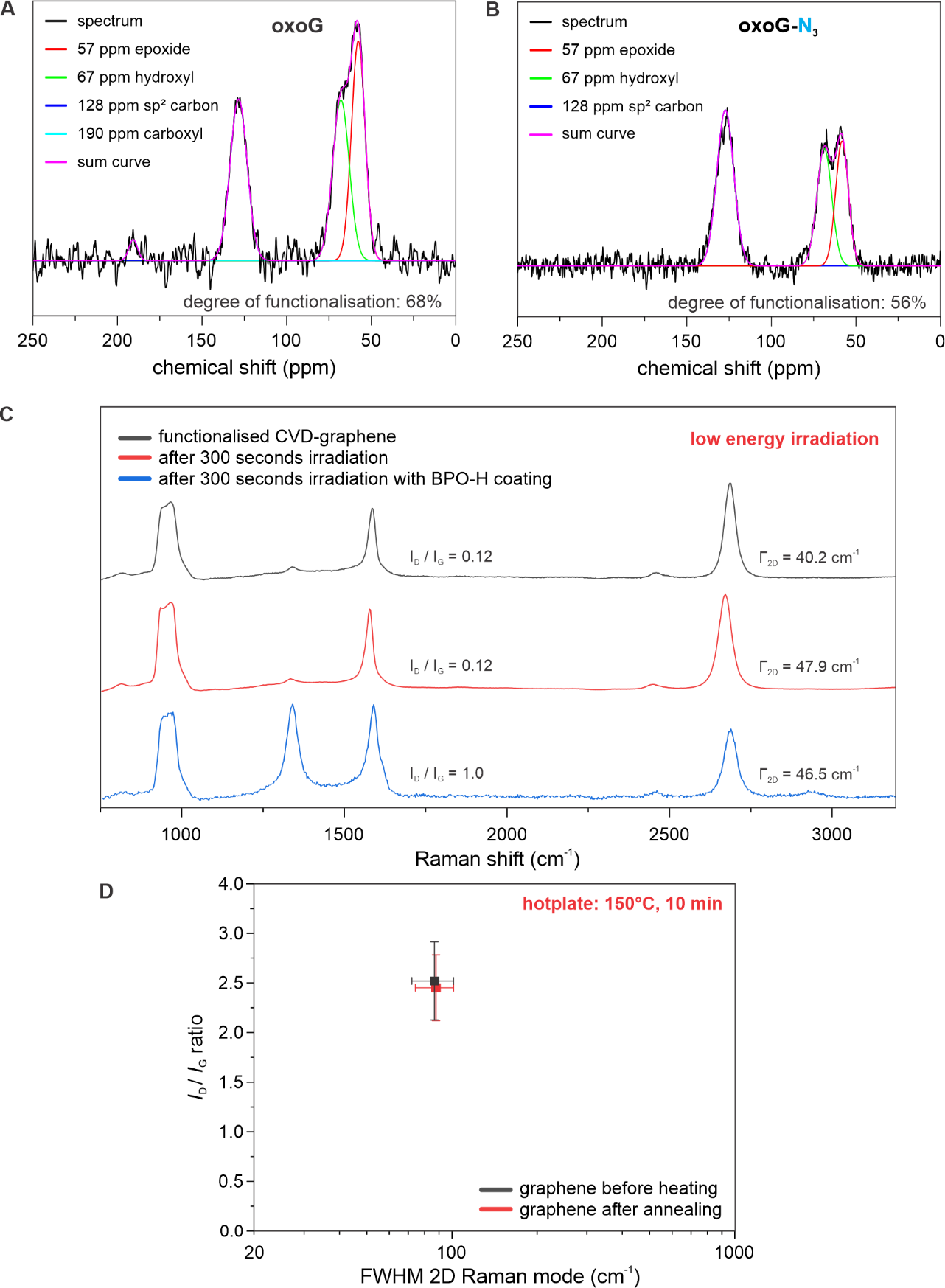
Figures

**Figure S1**: A) Solid state NMR spectrum of oxoG, prepared by wet-chemical oxidation of crystalline feed graphite and azide functionalized oxoG-N_3_. C) Raman spectra of CVD graphene (black), irradiated in the absence of BPO-H (red) and after dip-coating in BPO-H solution under similar conditions. The Raman spectra change only in the presence of BPO-H. D) Statistical analysis of the Raman spectra of graphene on 300 nm SiO_2_/Si wafer before and after annealing at 150°C for 10 minutes on a hotplate. It can be seen, that the Raman spectra do not change significantly in the absence of BPO-H. For the evaluation, Raman maps with >450 spectra were analyzed. Low-Energy: 0.31 mW, 1.0 sec acquisition time. High-Energy: 4.7 mW; 1.0 sec acquisition time.


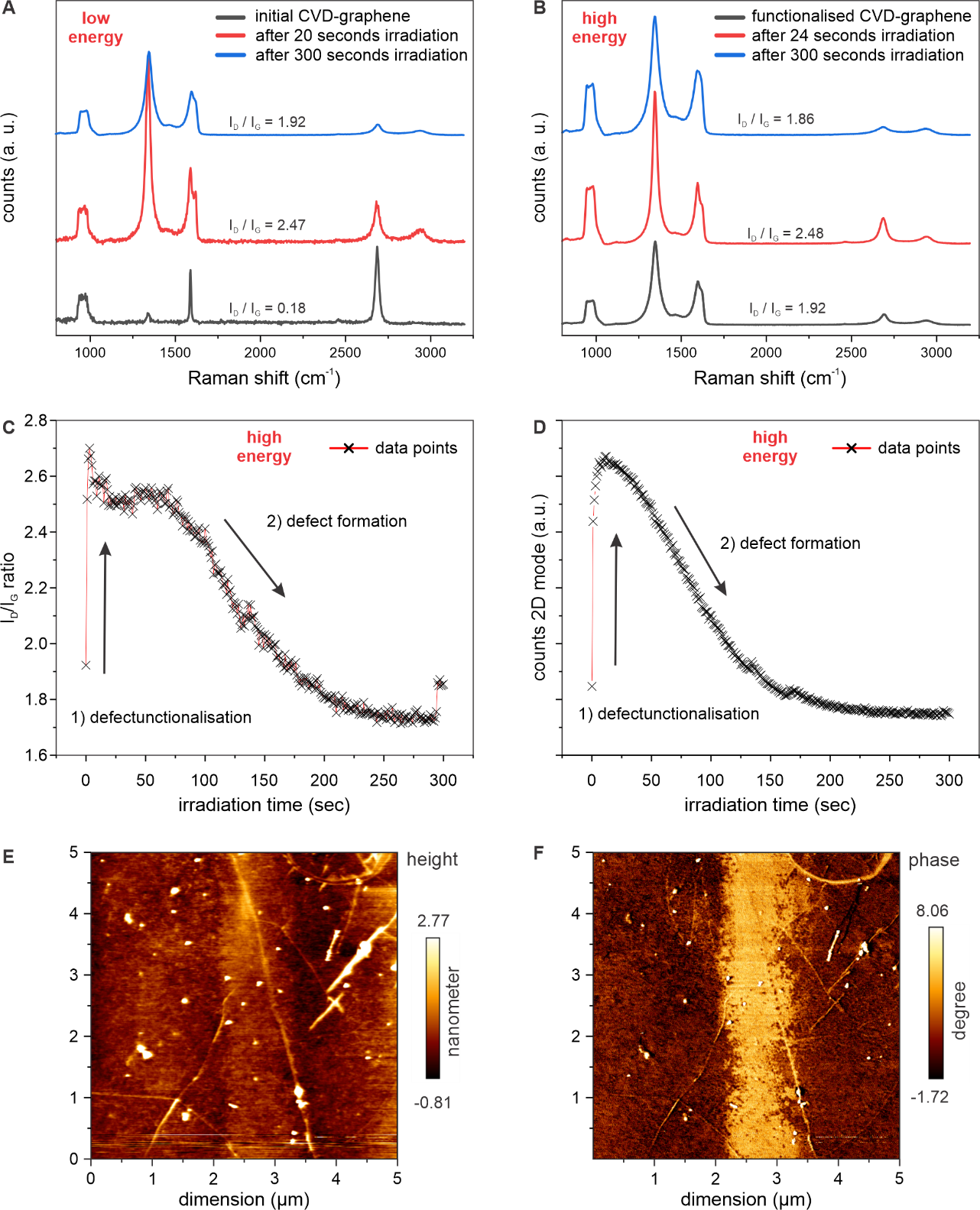


**Figure S2**: A) CVD graphene with BPO-H coating after specific times of irradiating with medium laser intensity (532 nm), leading to a functionalisati9on of the carbon lattice. B) Functionalized and washed CVD graphene coating after specific times of irradiating with high laser intensity (532 nm), leading to a defunctionalisati9on of the carbon lattice. C) Evolution of the I_D_/I_G_-ratio and D) 2D mode intensity over the course of 300 seconds. E, F) AFM height and phase images of the irradiated area on CVD graphene. Low-Energy: 0.31 mW, 1.0 sec acquisition time. High-Energy: 4.7 mW; 1.0 sec acquisition time.


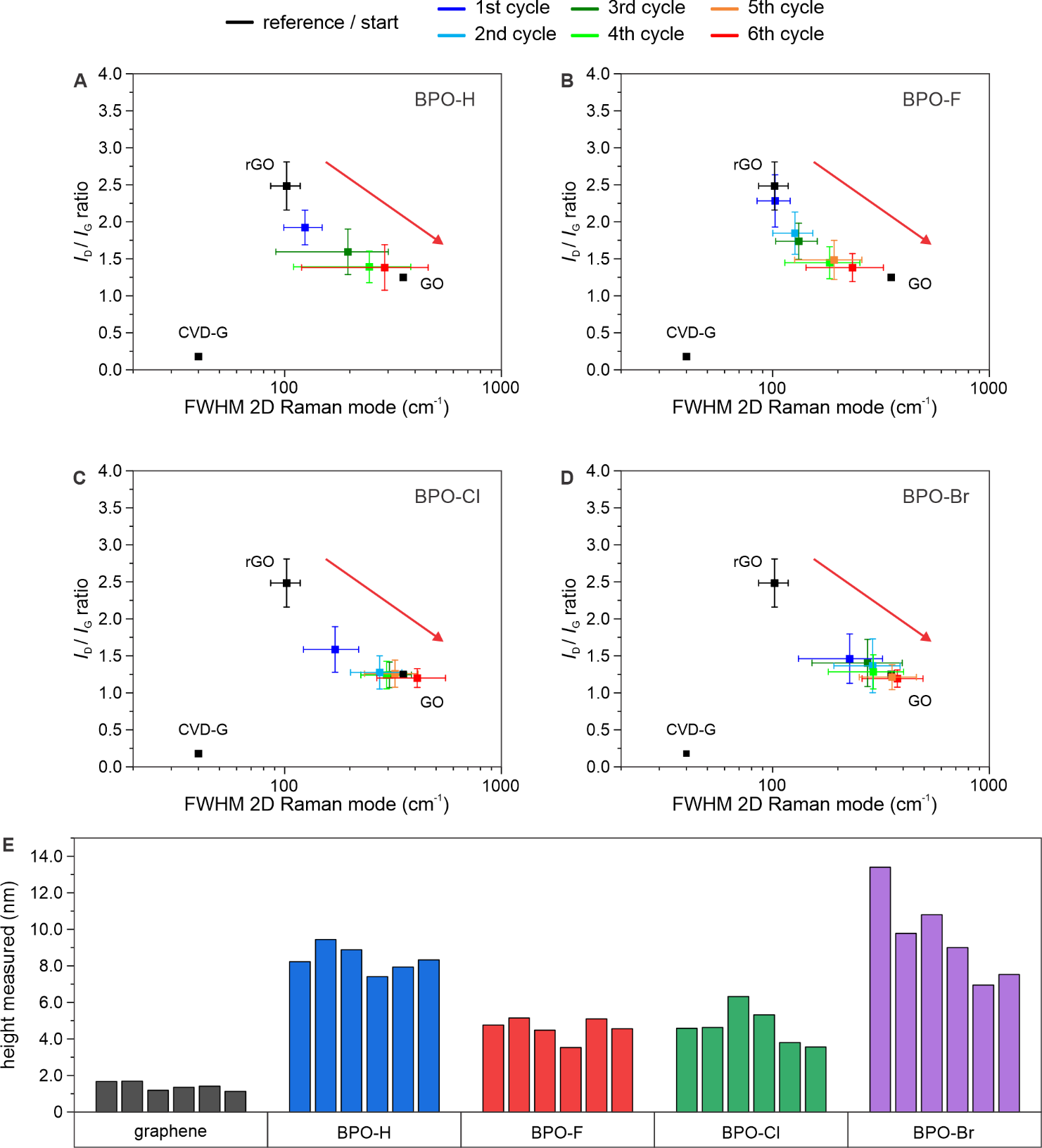


**Figure S3.** A-D) Statistical analysis of the spectra of recorded Raman maps: evolution of the *I_D_*/*I_G_* ratio and FWHM_2D_ with each cycle of thermal functionalzation with bezoyl peroxide derivatives. E) Extracted height values of (functioanlised) graphene flakes after 6 cycles of functionalization.


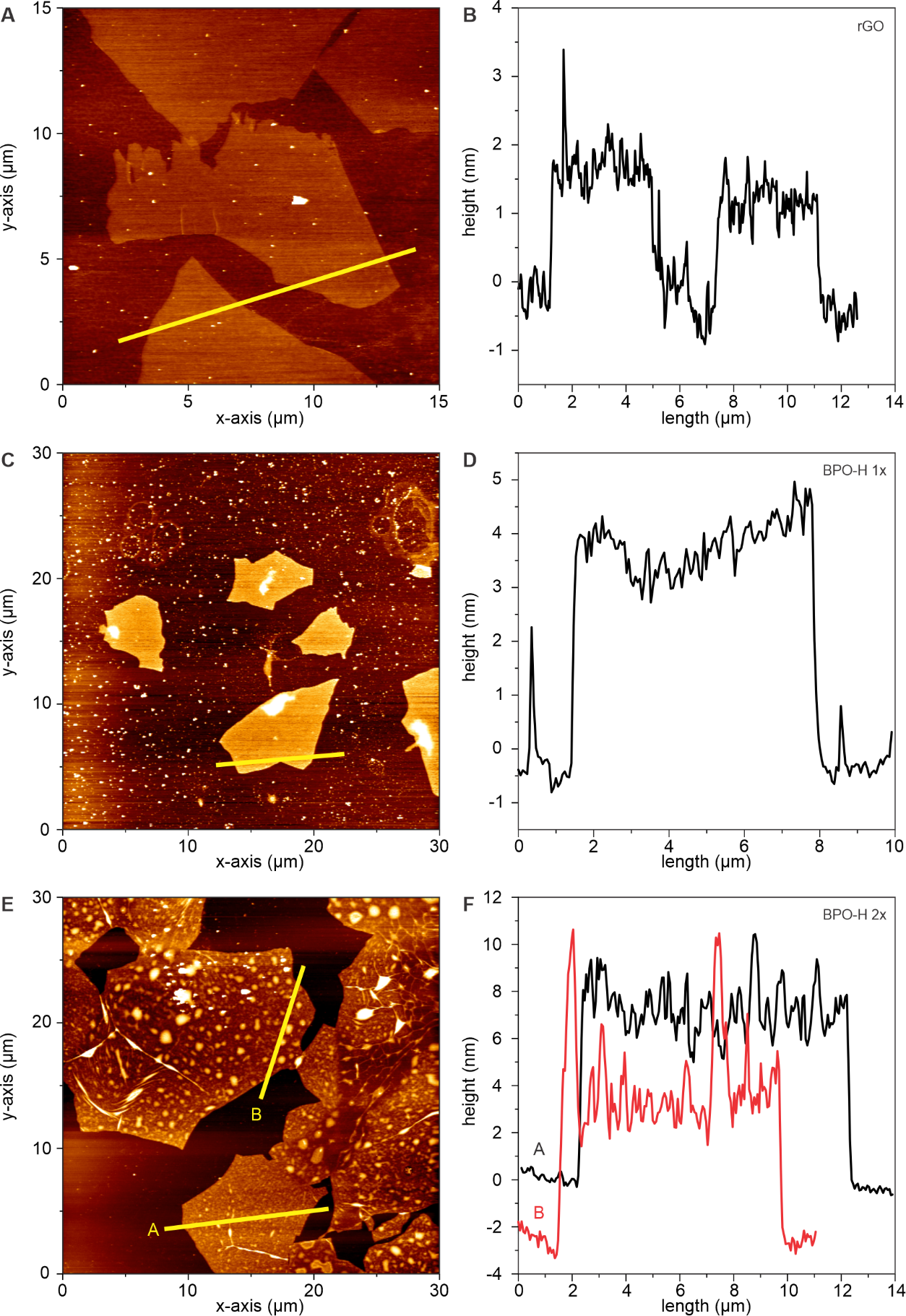


**Figure S4**: AFM height images together with the corresponding extracted height profiles of functionalized graphene samples. A+B) unfunctionalized graphene; C+D) graphene functionalized with BPO-H for one time; E+F) graphene functionalized with BPO-H for two times.


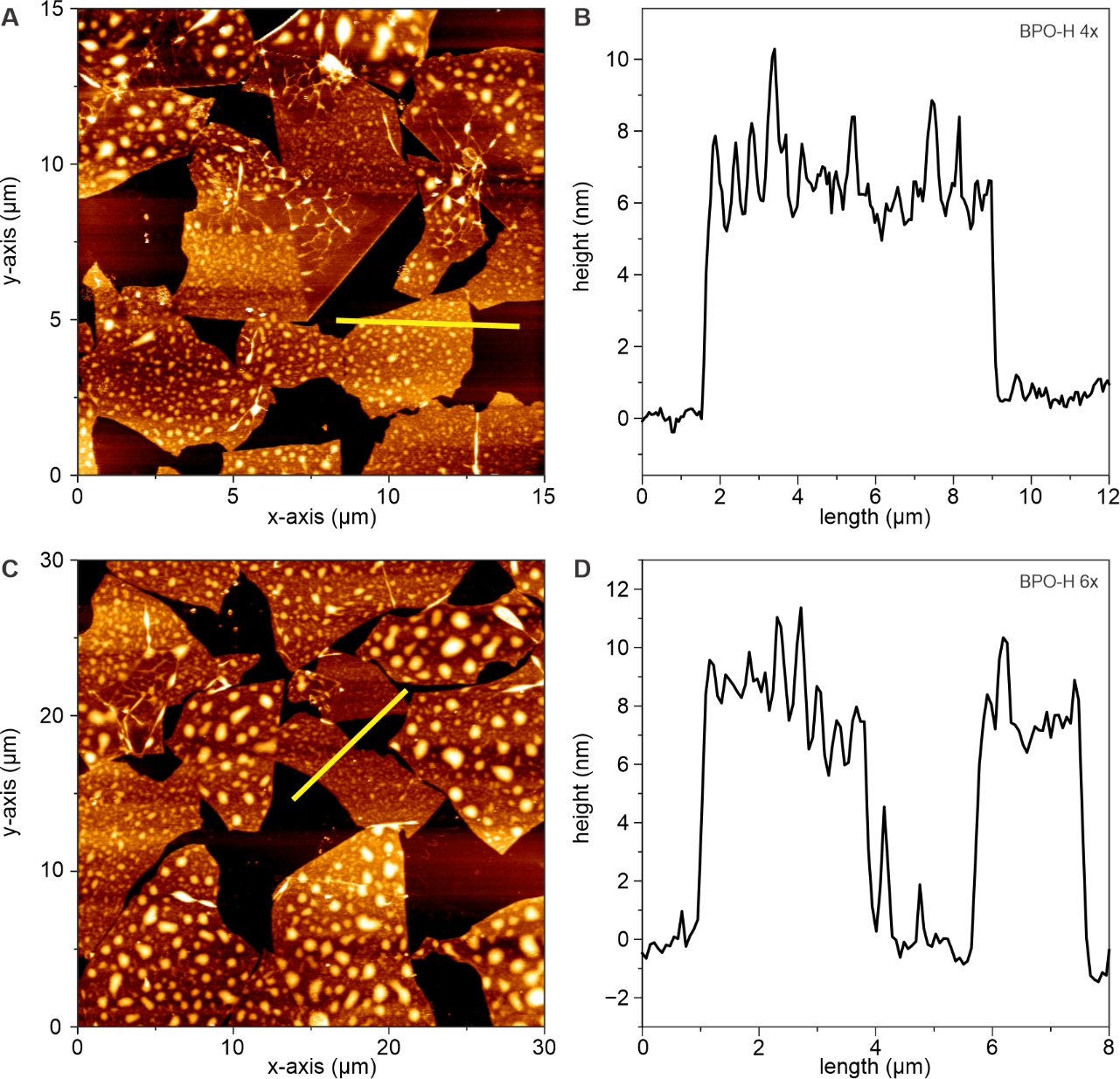


**Figure S5**: AFM height images together with the corresponding extracted height profiles of functionalized graphene samples. A+B) graphene functionalized with BPO-H for four times; C+D) graphene functionalized with BPO-H for six times.


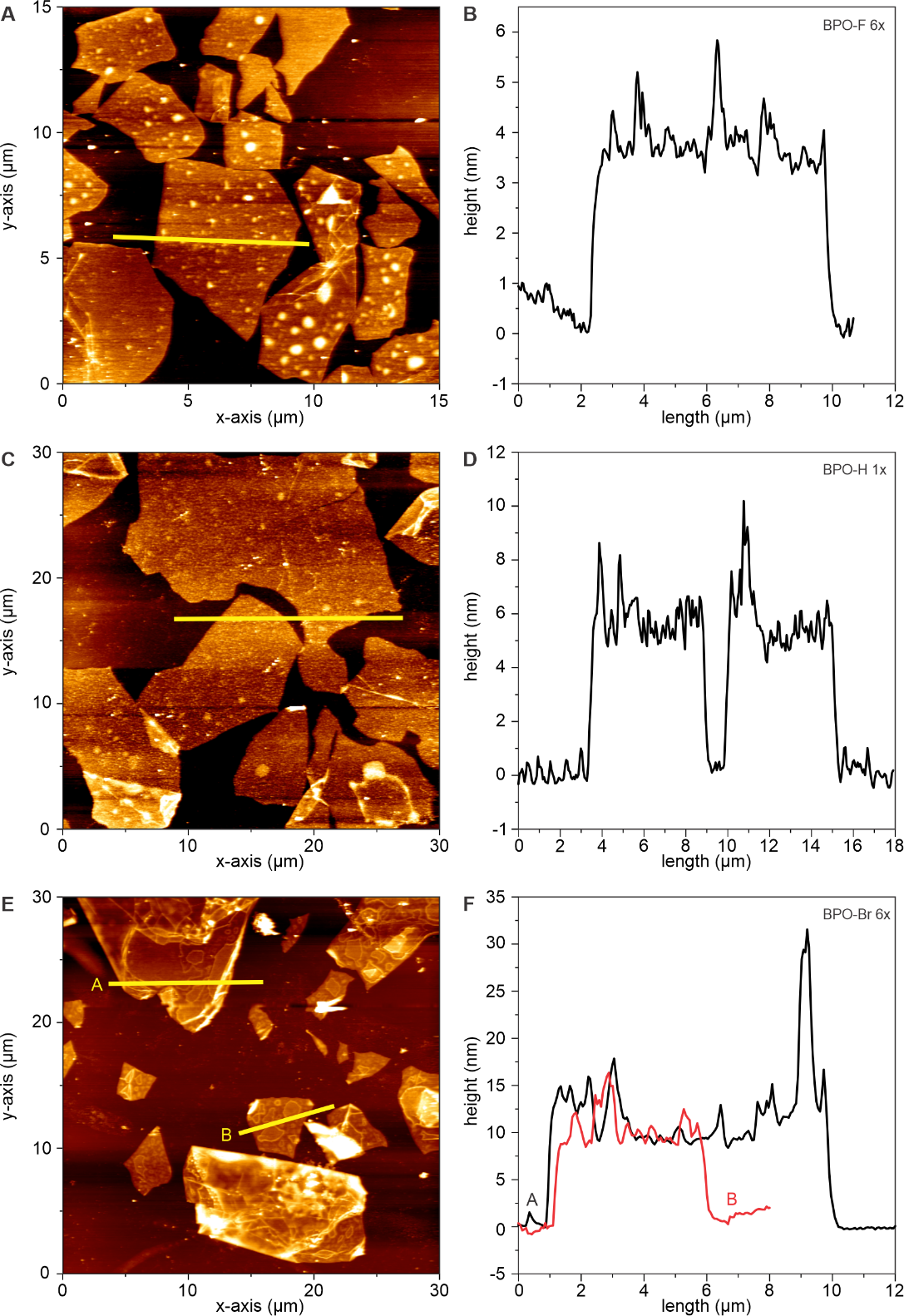


**Figure S6**: A) AFM height images together with the corresponding extracted height profiles of functionalized graphene samples. A+B) graphene functionalized with BPO-F for six times; C+D) graphene functionalized with BPO-Cl for six times; E+F) graphene functionalized with BPO-Br for six times.


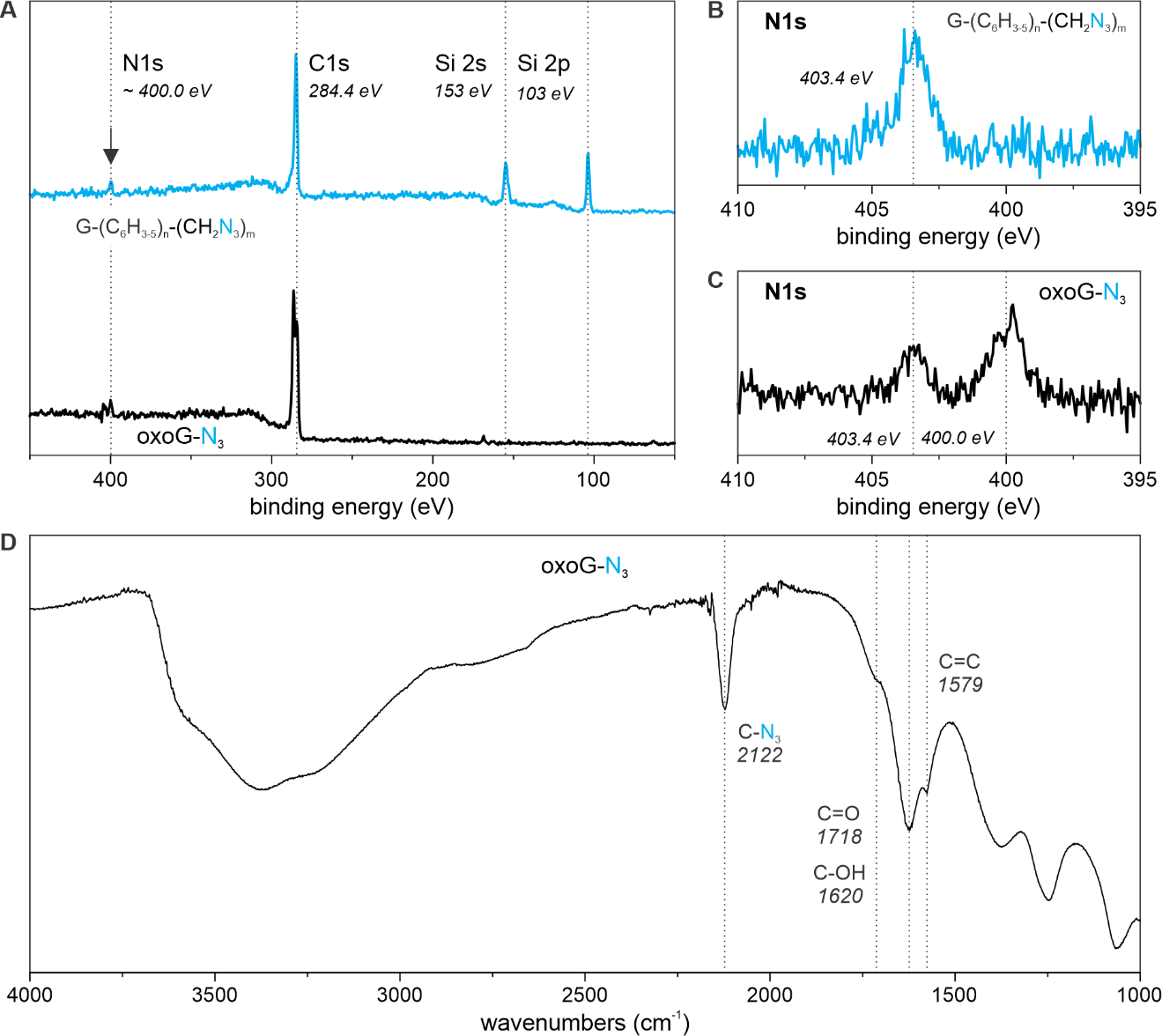


**Figure S7**: A) XPS survey and high resolution N1s core spectrum of the azide functional group an azide-functionalized Graphene with oligophenylene structures and pristine oxoG (oxoG-N_3_). B, C) Corresponding N1s high resolution core spectra. D) FTIR spectrum with a visible absorption band for covalently bound azide group on the carbon lattice of graphene.

Synthetic procedures

Chemicals were purchased from Merck/Sigma-Aldrich (Germany) and used as received unless otherwise stated. Double-distilled water was purchased from Carl Roth (Germany). Silicon substrates with a 300 nm thick SiO_2_ layer were purchased from Fraunhofer IIS (Erlangen, Germany) and cleaned with piranha solution before use.

Raman: Horiba XploRA Plus equipped with a green 532 nm laser (100 mW) and an automated XYZ stage from Märzhäuser Wetzlar (Germany). The laser power was adjusted with specific filters. An objective with 100x magnification was used for laser writing and analysis. Low-Energy: 0.31 mW, 1.0 sec acquisition time. High-Energy: 4.7 mW; 1.0 sec acquisition time. The Raman spectra were fitted with Lorentzian curves using a custom programmed python script and statistically analyzed using OriginPro 2024 (Origin Labs) ^[1]^. AFM: JPK Nonwizard 2 with Tap300Al-G cantilever (AC mode) from BudgetSensors (Bulgaria). Fluorescence: Perkin-Elmer FL-6500. Fluorescence maps were recorded in the range 200-600 nm (excitation) and 300-900 nm (emission). The emission slit was 2.5 nm, the excitation slit was 1 nm and the scan speed was 1200 nm/min. XPS: Specs EnviroESCA (source: Al K_α_: 1486.71 eV; PHOIBOS 150 electron energy analyzer). The spectra were measured in normal emission, and a source-to-sample angle 55° was used. All spectra were acquired in fixed analyzer transmission (FAT) mode. The binding energy scale of the instrument was calibrated, following a technical procedure provided by SPECS Surface Nano Analysis GmbH (ISO 15472). All binding energies were referenced to the signal observed for the aliphatic C–C bond component (Ebind = 285 eV) if not denoted otherwise. The spectra were quantified utilizing the empirical sensitivity factors that were provided by SPECS Surface Nano Analysis GmbH (the sensitivity factors were corrected with the transmission function of the spectrometer). Survey spectra: 100 eV pass energy and 0.5 eV step width. High resolution core spectra: 30 eV pass energy and 0.05 eV step width, 8 accumulations. The spectra obtained were processed with UniFIT 2022 ^[2]^.

Preparation of Peroxyanhydride-Derivatives

The halogenated peroxides were prepared from a reaction of the corresponding acid chlorides with hydrogen peroxide following the following general protocol. The halogenated benzoyl chloride (3.2 mmol, 1.6 eq.) was dissolved in diethyl ether (2 ml) and cooled down to 0°C. A 30% hydrogen peroxide solution (68.0 mg, 2.0 mmol, 1 eq.) was added dropwise over a period of 10 minutes to the 4-halobenzoyl chloride. The reaction was stirred for a further 10 minutes. Afterwards sodium hydroxide (160.0 mg, 3.90 mmol, 1.95 eq.) was dissolved in 2 ml of water and added over a period of 20 minutes to the 4-halobenzoyl chloride solution. The solution was filtered off and the precipitate was washed several times with cold acetone, water and diethyl ether to obtain a white powder.

4-Bromobenzoic Peroxyanhydride (BPO-Br)

4-bromobenzoyl chloride (702.3 mg, 3.2 mmol, 1.6 eq.) was dissolved in diethyl ether (2 ml) and cooled down to 0°C. A 30% hydrogen peroxide solution (68.0 mg, 2.0 mmol, 1 eq.) was added dropwise over a period of 10 minutes to the 4-bromobenzoyl chloride. The reaction was stirred for a further 10 minutes. Afterwards sodium hydroxide (160.0 mg, 3.90 mmol, 1.95 eq.) was dissolved in 2 ml of water and added over a period of 20 minutes to the 4‑bromobenzoyl chloride solution. The solution was filtered off and the precipitate was washed several times with cold acetone, water and diethyl ether to obtain a white powder (59.6%).

^1^H NMR (400 MHz, Chloroform-*d*) δ 7.95 – 7.91 (m, 4H), 7.69 – 7.65 (m, 4H). R_f-_value = 0.4 (4:1 hexane/ethyl acetate) [UV].

The spectroscopic data obtained are in agreement with those previously reported.^[3]^

4-Fluorobenzoic Peroxyanhydride (BPO-F)

4-fluorobezoyl chloride (634.2 mg, 4.0 mmol, 1.6 eq.) was dissolved in diethyl ether (2.5 ml) and cooled down to 0°C. A 30% hydrogen peroxide solution (85.0 mg, 2.5 mmol, 1 eq.) was added dropwise over a period of 10 minutes to the 4-fluorobezoyl chloride. The reaction was stirred for a further 10 minutes. Afterwards sodium hydroxide (195.0 mg, 4.9 mmol, 1.95 eq.) was dissolved in 2.5 ml of water and added over a period of 20 minutes to the 4‑fluorobezoyl chloride solution. The solution was filtered off and the precipitate was washed several times with cold acetone, water and diethyl ether to obtain a white powder (66%).

^1^H NMR (400 MHz, Chloroform-*d*) δ 8.14 – 8.07 (m, 4H), 7.24 – 7.17 (m, 4H). R_f-_value = 0.72 (9:1 hexane/ethyl acetate) [UV].

The spectroscopic data obtained are in agreement with those previously reported.^[3]^

4-Chlorobenzoic Peroxyanhydride (BPO-Cl)

4-chlorobenzoyl chloride (700.0 mg, 4.0 mmol, 1.6 eq.) was dissolved in diethyl ether (2.5 ml) and cooled down to 0°C. A 30% hydrogen peroxide solution (85.0 mg, 2.5 mmol, 1 eq.) was added dropwise over a period of 10 minutes to the 4-chlorobenzoyl chloride. The reaction was stirred for a further 10 minutes. Afterwards sodium hydroxide (195.0 mg, 4.9 mmol, 1.95 eq.) was dissolved in 2.5 ml of water and added over a period of 20 minutes to the 4-‑chlorobenzoyl chloride solution. The solution was filtered off and the precipitate was washed several times with cold acetone, water and diethyl ether to obtain a white powder (65.3%).

^1^H NMR (400 MHz, Chloroform-*d*) δ 8.03 – 7.99 (m, 4H), 7.52 – 7.48 (m, 4H). R_f-_value = 0.62 (9.5:0.5 hexane/ethyl acetate) [UV].

The spectroscopic data obtained are in agreement with those previously reported.^[4]^

Preparation and Handling Graphene-Based Samples

CVD-Graphene

Low defective Graphene was prepared by chemical vapor deposition on copper foil folded into an envelope. The copper envelope was annealed for 1 h (1035°C, 10 sccm H_2_, 5 sccm Ar), then methane (5 sccm) was added to the mixture for 10 min. The sample was quickly cooled to room temperature. Finally, pieces of graphene were transferred to the substrates using the wet transfer method. Raman: CVD-G: *I*_D_/*I*_G_ < 0.2.

Oxo-functionalized Graphene (oxoG)

A dispersion of oxoG was prepared by wet chemical oxidation of graphite (3 g) in concentrated sulphuric acid (120 mL) with potassium permanganate (9 g). The oxidant was added slowly over 4 hours and stirred overnight. The reaction mixture was quenched by slow addition of 20 % sulphuric acid (100 mL), pure water (100 mL) and hydrogen peroxide (5 %, 100 mL). The dispersion obtained was purified by repeated centrifugation to a pH of ~6-7. Exfoliation was performed using a tip sonicator (total 4 min, 40 watts, pulse 1 sec on, 1 sec off). The material was further purified by gentle centrifugation at low RFC to remove non-exfoliated material four times and finally at high RFC to remove impurities and very small diameter particles. EA: C: 63.5%; H: 1.9%; N: <0.01; S: 4.7%. ssNMR: C(epoxy) = 57 ppm, C(hydroxy) = 67 ppm, C(sp²) = 128 ppm, C(carboxy) = 190 ppm; degree of functionalization = 68%. Raman: oxoG: *I*_D_/*I*_G_ = 1.25.

Azide-Functionalized oxoG

Azide functionalized oxoG was prepared by mixing 50 mg of the nanomaterial with 100 mg sodium azide in 200 mL of water for 10 minutes before the dispersion was subsequently lyophilized. After complete sublimation overnight, the material was purified by repetitive centrifugation. EA: C: 41.5%; H: 9.7%; N: 3.2%; S: 0.0%. ssNMR: C(epoxy) = 57 ppm, C(hydroxy) = 67 ppm, C(sp²) = 128 ppm, C(carboxy) = 190 ppm; degree of functionalization = 68%.

Reduction of oxo-functionalized Graphene to Graphene

Reduction of oxoG on SiO_2_/Si substrates was carried out by placing a wafer in a glass vial on glass wool. A drop of hydrogen iodide (57%) and a few drops of trifluoro acetic acid (99.5%) were then added. The vial was sealed with a perforated plastic cap and heated at 100°C for 5-10 minutes. The wafer was then carefully rinsed with double distilled H_2_O and dried with a nitrogen gun. Raman: reduced oxoG: *I*_D_/*I*_G_ ~ 3.39 (selected flakes); statistical batch analysis *I*_D_/*I*_G_ = 2.48 ± 0.33).

Functionalization of Graphene with BPO-R

Functionalization of graphene was conducted by immersing 300 nm SiO_2_/Si wafer (5x5 mm) with a film of single layer graphene on the surface for 5 seconds in a solution of the organic peroxide in acetone or dichloromethane (5 mL, ~ 5 mM). The remaining organic solvent was evaporated under ambient conditions. Ultimately, the wafer was placed on a clean hotplate for 1 minute at 150°C and washed with clean solvent to form oligophenylene structures on the graphene surface. The process was repeated up to six times.

Bromomethylation of Graphene with Covalent Oligophenylene-Structures

Bromomethylation was achieved by immersing oligophenylene functionalized graphene on 300 nm SiO_2_/Si wafer into a solution of glacial acetic acid and 55 % hydrogen bromine in acetic acid. The wafers were carefully washed for at least 3 times by immersing the solid substrates for 1 hour in excess of clean water.

Secondary functionalization of bromomethylated Graphene with Covalent Oligophenylene-Structures

Nitrile and Azide groups were introduced by immersing bromomethyl functionalized materials in aqueous solutions at room temperature for 24 h. The wafers were carefully washed for at least 3 times by immersing the solid substrates for 1 hour in excess of clean water.

References

[1] S. Eigler, F. Hof, M. Enzelberger-Heim, S. Grimm, P. Müller, A. Hirsch, *J. Phys. Chem. C* **2014**, *118*, 7698-7704. 10.1021/jp500580g

[2] T. R. Gengenbach, G. H. Major, M. R. Linford, C. D. Easton, *Journal of Vacuum Science & Technology A* **2021**, *39*. Artn 013204

10.1116/6.0000682

[3] S. Rajamanickam, C. Sah, B. A. Mir, S. Ghosh, G. Sethi, V. Yadav, S. Venkataramani, B. K. Patel, *J. Org. Chem.* **2020**, *85*, 2118-2141. 10.1021/acs.joc.9b02875

[4] N. Yadav, S. R. Bhatta, J. N. Moorthy, *J. Org. Chem.* **2023**, *88*, 5431-5439. 10.1021/acs.joc.2c03059
